# Practical applications of teledentistry during the Covid-19 pandemic in ASEAN member states – a systematic review

**DOI:** 10.1186/s12903-024-04177-x

**Published:** 2024-04-05

**Authors:** Mandy Loh Jin-Yu, Cheong Wayn Min, Jason Law Si Jin, Muneer Gohar Babar, Syed Sarosh Mahdi

**Affiliations:** Division of Clinical Oral Health Sciences, School of Dentistry, International Medical University, Kuala Lumpur, Malaysia

**Keywords:** Teledentistry, Dentistry, ASEAN, Digital health, Dental health, Telemedicine

## Abstract

**Objective:**

The objective of this review is to determine the utilisation and adoption of teledentistry based solutions and technologies during the Covid-19 Pandemic in the Asean region.

**Background:**

Teledentistry is a branch of telemedicine that has rapidly advanced in the last few years and has the potential to provide solutions to oral health problems of patients and locations that do not have prompt and immediate access to a dentist or dental services. The Covid-19 has increased the adaption of all digital health technologies and teledentistry is no exception.

**Methodology:**

The study utilized online databases such as Pubmed (Medline), Scopus (Embase) and CINAHL for the purpose of document search. Newcastle Ottawa (NOS) scale was used to determine the quality of the studies included in our systematic review. PRISMA guidelines were used as the criteria for reporting items in the systematic review.

**Results:**

A total of 1297 documents were found after applying the search criteria and the keywords for the selected study. After applying the Prisma guidelines, removal of duplicates and irrelevant entries, 10 studies that were conducted during the Covid-19 pandemic were selected, fitting the inclusion criteria. All the studies included were evaluated for quality and risk of bias through the Newcastle Ottawa scale. Only high-quality studies were included for the final review.

**Conclusion:**

Teledentistry is a cost-effective solution to screen, diagnose and treat dental patients from a distance. Teledentistry also has the potential to continue seamless continuation of dental education to dental students, during disruptive and non-disruptive periods. ASEAN countries should fully utilise the potential of teledentistry, however sound and effective legislation would be the key first step to achieving that potential.

## Introduction

Telemedicine, otherwise known as telehealth, is a rapidly developing field that utilises information and communication technologies (ICT) to deliver healthcare services over long distances. Introduced in the 20th century, e-health services were initially adopted mainly by developed nations. However, with advances in ICT and improved access to technological resources in recent years, telemedicine has been gaining popularity even in developing countries, changing the healthcare landscape in developing nations [1]. Furthermore, the Covid-19 outbreak has served as a catalyst, prompting healthcare professionals worldwide to embrace telehealth services [2–4]. While telemedicine is readily embraced worldwide, its dental counterpart is severely lacking, especially among developing nations.

On 11 March 2020, four months after the first case of COVID-19 was detected in Wuhan, China, the World Health Organization (WHO) had officially announced it as a pandemic [5]. COVID-19, caused by a novel strain of Coronavirus named SARS-CoV-2, spreads through respiratory droplets, direct contact or through fomites. As of the 31st of May 2023, a staggering 767,364,883 cases of Covid-19 had been reported globally, with a death toll of 6,938,353 [5]. Although the WHO had rescinded its pandemic status in early May 2023 [5], more than three years since COVID-19 was declared a global emergency, it has left a profound effect on the world.

At the start of the pandemic, elective dental procedures were postponed as per instructions from the WHO to mitigate cross-infection risks [6]. The proximity between patients and dentists, as well as the aerosol-producing nature of most procedures, creates a conducive environment for the virus to spread. When lockdowns were enforced, dental practice was brought to a virtual halt. The stringent measures imposed greatly reduced the public’s access to proper dental services during this period. Decreased access to proper health care, corona-phobia, bad dietary habits and altered oral health behaviours led to a reduction in oral health status during this crisis [7–9]. According to Lyu et al. [10], parents in the United States believed that their children’s oral health deteriorated during the pandemic. Furthermore, from the same study, the incidence of gingivitis among children saw a sharp rise in 2020. This article showed that Covid-19 had an adverse effect on oral health. Similar results had been reflected in the United Kingdom, Israel, Australia, Italy, Chile and Brazil [9–11].

To overcome the challenges of limited access to dental services, teledentistry was the most viable alternative during the pandemic. There was a mushroom growth of telehealth-based services, especially in the ASEAN region during the COVID-19 outbreak [12]. An article by Sycinska-Dziarnowska et al. [13] had also detected a sudden spike in the search term “teledentistry” at the start of the pandemic, in levels that far surpass the pre-pandemic era. Telehealth services are no stranger in the field of dentistry, with its first utilisation dating back in 1994 as part of the US Army’s Total Dental Project [14]. Teledentistry is a branch of telehealth where information technology is used to provide dental services at a distance, hence eliminating the need for traditional face-to-face interactions. Teletriaging, teleconsultation, online referral, tele-treatment, tele-education, tele-monitoring and tele-surgery are among the services provided under the umbrella of teledentistry [15]. Telehealth services have been proven to be a cost-effective measure to reduce health care inequalities [16, 17]. Although teledentistry is potentially beneficial in times of emergencies, some health care workers are reluctant to embrace it due to various reasons. Among them were limited technology know-how, incompetency, costs, privacy issues and quality of intraoral pictures [15, 18].

The literature on teledentistry in the ASEAN region is limited, and this systematic review aims to fill in some gaps in the knowledge present on this subject, particularly on its effectiveness, how widespread it was being implemented, and areas of dentistry where e-health services were most often used during the COVID-19 pandemic.

## Methodology

### Study protocol and registration

A study protocol was created, based on the Preferred Reporting Items for Systematic Reviews and Meta-Analyses Protocols (PRISMA) [19]. The authors registered the systematic review with the Prospective Register of Systematic Reviews (PROSPERO), University of York. The study was allocated the registration number CRD42023397668.

### Document search

In order to find pertinent publications published between January 2020 and July 2023, three investigators independently carried out an electronic search using three online databases which included, Cumulative Index to Nursing and Allied Health Literature (CINAHL), Scopus, and PubMed. Keyword ‘Teledentistry’, ‘COVID-19’, and ‘ASEAN countries’ with relevant Medical Subject Headings (MeSH) terms were applied equally for each search in each database along with the use of Boolean operators such as ‘AND’ and ‘OR’. (Table 1) The references of each qualified paper were also scanned through for other papers which were relevant to our title, that may have been missed out during the search. In cases where a consensus was not reached, opinion was sought from the fourth reviewer.

**Table 1:** Search terms and databases

Databases	PubMed, CINAHL, Scopus	
Search terms	Teledent*	ASEAN
	Tele-diagnosis	Southeast* Asia*
	Remote dent*	Brunei*
	Virtual dent*	Cambodia*
	E-Dent*	Indonesia*
	mHealth	Lao*
	Mobile health	Malaysia*
	Dent* telecomunicat*	Philippines
	Oral telehealth	Filipino*
	E-oral health	Singapore*
	Online dent* consult*	Thai*
	Online dent* diagnos*	Vietnam*
	Online refer* system	Myanmar*
	Online oral med	Burm*
MESH terms	Virtual med*	Borneo
	Tele* refer*	Brunei
	Mobile health	Cambodia
		Indonesia
		Laos
		Malaysia
		Myanmar
		Philippines
		Singapore
		Thailand
		Vietnam

### Inclusion/exclusion criteria

A full-text examination was carried out by three researchers to find English-language publications that had at least one keyword that is related to the title. The articles in the final review were those that were published in highly reputable, indexed & established journals. The timeframe for our search strategy included articles from January 2020 to July 2023, which was during the COVID-19 pandemic period.

The exclusion criteria the study employed excluded narrative reviews, short communications, editorials, and letters to the editors. The authors also excluded Teledentistry based studies that were conducted in countries, other than ASEAN countries. Articles that were published prior to the Covid-19 pandemic but published during the abovementioned period were removed. The final review also excluded articles that were published in non-peer reviewed and non-indexed journals. (Table 2)

**Table 2:** Table showing the inclusion and exclusion criteria

Inclusion criteria	Exclusion criteria
Articles published in indexed journals	Articles published in non-indexed journals
Articles written in English	Articles written in any languages other than English
Original articles on teledentistry usage during the Covid-19 pandemic, published within 1st January 2020-31st July 2023	Studies conducted before the Covid-19 pandemic but published within 1st January 2020- 31st July 2023
Studies done in the ASEAN region	Review articles, short communications, editorials, letters
	Studies not done in ASEAN nations

### Data extraction

After removal of duplicates using the PRISMA tool, three investigators performed a full text assessment of each remaining article for eligibility. Parameters such as ‘subunits of Teledentistry’, ‘ASEAN country’, ‘type of article’ and ‘domain’ were identified, and the results were categorised in a customised google spreadsheet.

### Quality assessment

The assessment of bias is a crucial aspect while carrying out a systematic review. Our study integrated the Newcastle-Ottawa scale (NOS) (Table 3) to identify the rigorousness and strength of the studies as well as bias assessment [20]. The Newcastle Ottawa scale grades the included articles in the review by assigning stars to various domains of quality assessment. NOS scale is a 9- or 10-stars scale and the higher number of stars denotes higher quality of research and less bias. NOS scale rate the standard of the studies in the review on critical aspects i.e. (comparability, outcome & selection). Interpreting the NOS scale is straightforward as the studies are graded as poor, fair and good with 0–4*, 5–6*, and 7–10* respectively for each grade.

**Table 3:** Newcastle-Ottawa scale quality assessment form for non-randomized studies included in the review

Study	1 2 3 4 5	6 7	8 9 10	Score *
Rajendran et al. 2023 [21]	* * *	*	* *	7
Khokhar et al. 2021 [22]	* * *	* *	* * *	8
Soegyanto et al. 2022 [23]	* * * *	* *	*	7
Amtha et al. 2022 [24]	* * * *	* *	* *	8
Maqsood et al. 2021 [25]	* * * *	* *	* * *	9
Santipipat et al. 2023 [26]	* * * *	* *	* * *	9
Roslan et al. 2023 [27]	* * * *	* *	* *	8
Amir et al. 2020 [28]	* * * *	* *	* * *	9
Pithpornchaiyakul et al. 2022 [29]	** *	* *	* * *	8
Zain et al. 2023 [30]	* * * *	* *	* *	8

## Results

### Search outcomes

**Fig. 1:**
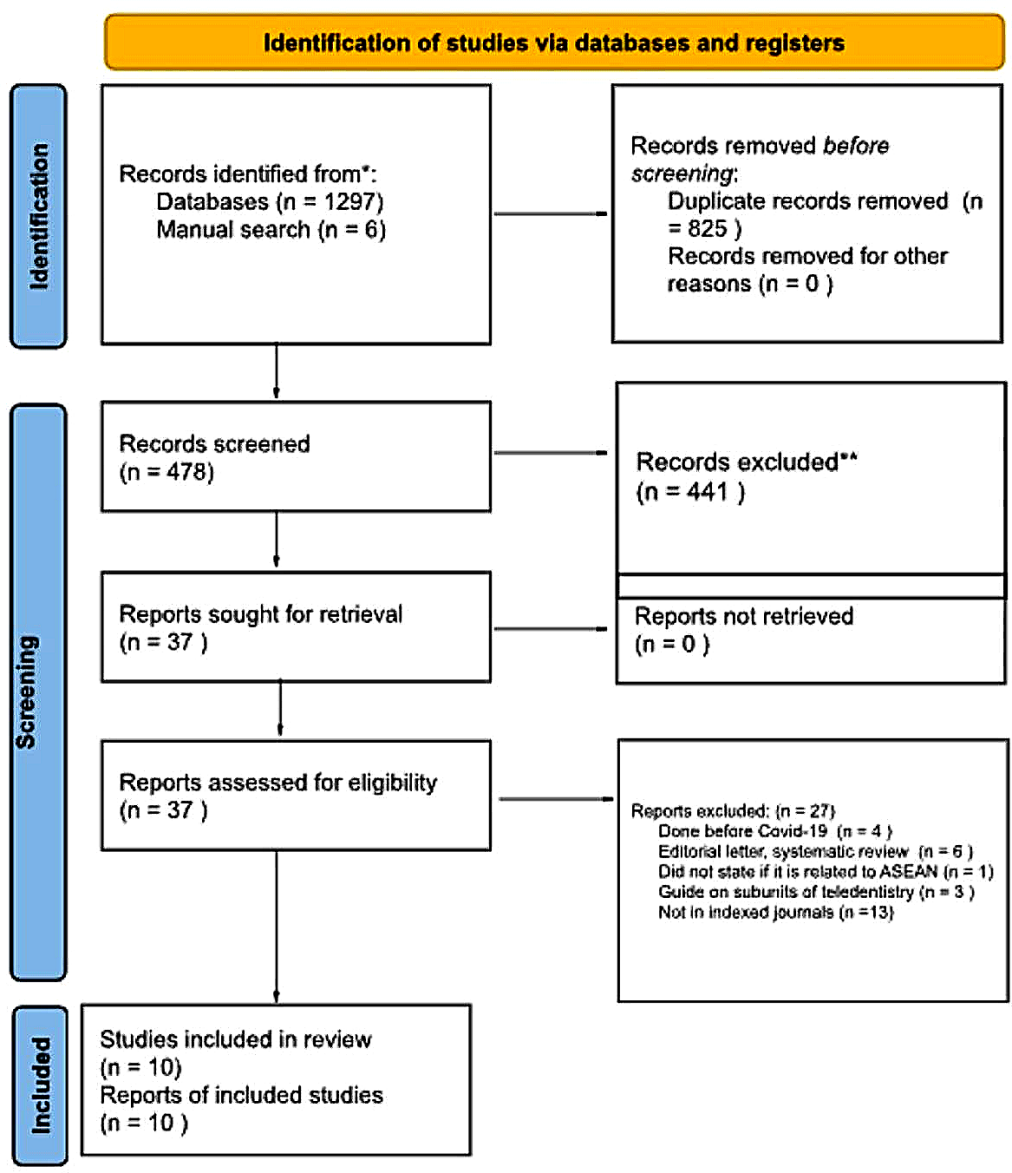
PRISMA flowchart for study selection

During the initial article search conducted by the authors, a total of 1297 papers were identified and retrieved from selected databases and 6 papers were identified by manual search. However, a total of 825 duplicate papers were identified and had to be removed. This was then followed by a thorough screening using the application of the inclusion and exclusion criteria which led to the elimination of 441 papers. 37 remaining articles were sought for retrieval and were chosen for full-text evaluation. 27 more papers had to be excluded after evaluation because they failed to meet the inclusion and exclusion criteria. Finally, after several discussions, 10 articles were selected to be included in this systematic review. Figure 1 shows the PRISMA flowchart used for the study selection and Table 4 shows the summary of study characteristics and findings.

**Table 4:** Summary of study characteristics and findings

N	Study type	Country	Participants	Teledentistry subtype	Design	Strength and weaknesses	References
1	Web interface development	Malaysia	MeMoSA® UPLOAD- 2703 images were uploaded between June 2019 to September 2020, MeMoSA® ANNOTATE − 7 specialist	Tele-diagnosis Teleconsultation	MeMoSA® UPLOAD and MeMoSA® ANNOTATE are the two components that make up the platform and all components are located on a secure cloud server, MeMoSA® Data Vault which can be accessed by authorised users by using a unique password provided to them. The function of MeMoSA® UPLOAD was for users to any images to the platform and MeMoSA® ANNOTATE allows for systemic annotations of the images. A survey was conducted to gather opinions regarding the user interface, the viability of the annotation process, and the accuracy of clinical descriptors.	Strengths - Sensitivity was high in providing the correct referral decision for cancer surveillance Weaknesses - Some images were out of focus - Some lesions were identified to be difficult to assess based on images alone - The research was only done in Asia. The researchers were not sure how it can establish a global network - Not enough comprehensive clinical information such as patient’s chief concern, medical history, oral hygiene products	Rajendran al. (21)
2	Cross-sectional survey study	Malaysia	310 Malaysian Dental Practitioners	Tele-consultation Telediagnosis Tele-education Tele-monitoring	Between May and July of 2021, a prevalidated electronic questionnaire was distributed to different dental practitioners in Malaysia. The questionnaire’s first section addressed demographic data in addition to favoured techniques of communication. The questionnaire’s second section was composed of Likert-type questions, each with a maximum of five points. There were 26 questions in total which were further separated into four headings: Practitioners’ data security, advancements in dentistry through teledentistry, the advantages it offers dental patients between May and July 2021, and how beneficial teledentistry is for dental practices.	Strength - A pre-validated questionnaire was used - Randomized retrieval of subject’s e-mail from the Malaysian Dental Council (MDC) database - The study showed that Malaysian dental professionals have a positive perception of Teledentistry as a concept. Weakness Significantly high number of questions in the questionnaire - Poor response rate - The usage of a 5-point Likert scale, since numerous studies have shown that responders are more likely to choose the medium value as a safe, moderate option than any value on either extreme due to psychological obstacles.	Khokhar et al. (22)
3	Cross-sectional survey-based study using a non-probability sampling method	Indonesia	652 dentists from different provinces	Teleconsultation Telediagnosis Telemonitoring	An electronic survey collecting information on demographics, the impact of teledentistry in improving dental practice for both the dentists and patients, and cyber-risks related to teledentistry was disseminated by Whatsapp from January to February 2021. This survey also explored the dentist’s preferred dental specialties to be conducted virtually	Strengths: - The first study in Indonesia that explored the perception of teledentistry implementation by dentists, as well as the elements that may affect their views - Findings will aid future policy making Weakness: - Survey is not randomised - Presence of response bias	Soegyanto et al. (23)
4	Observational cross-sectional study	Indonesia	Patients from outpatient Clinic of Dental Hospital, Faculty of Dentistry Universitas Trisakti, who receivied teledentistry based services during the Covid-19 pandemic were included in the study.	Teleconsultation Telediagnosis	The level of patient satisfaction on teledentistry services were gauged via a validated questionnaire	Strength: - A pre-validated questionnaire was used, increasing the reliability and accuracy of the data collected - A Cronbach’s alpha score of 0.83 and a reliability item of 0.95 with a separation of 4.49 was obtained. Weakness: - Insufficient sample size	Amtha et al. (24)
5	Descriptive Observational Study	Malaysia, Singapore, Indonesia	This observational study had 506 participants in total	Teleconsultation, Telediagnosis, Telemonitoring, Tele-education	Between June and July of 2021, a verified electronic questionnaire was distributed via email and several social media platforms to the dental practitioners who were chosen. The questionnaire’s first section asked about demographics, communication method preferences, and professional background. Likert-type responses on a five-point scale were used for the second section of the questionnaire. The statistical analysis was conducted using the SPSS-25. The frequency, percentage, mean, and standard deviation of the demographic characteristics, the participant’s qualification, experience, practice location, and the use of teledentistry in various specialties were all assessed using descriptive statistics. The effect of independent variables (age, gender, education, and years of experience) on dependent variables (teledentistry domains) was examined using the ANOVA test. A statistically significant result was defined as a p value of less than 0.05.	Strength - Inclusion of dentists globally Weakness - Self-reported biasness. - Dentists working in the rural areas were not included in the study (can be considered which could provide a better view of teledentistry in such localities.)	Maqsood et al. (25)
6	Randomized control trial	Thailand	8 male prisoners - phase 1 309 male prisoners - phase 2 152 male prisoners for phase 3	Tele-diagnosis	There were a total of three phases in the dental disease screening program. Phase I: (PHVs) (Prison health volunteers) were given a teledentistry training program. Phase II: Oral Health screening was conducted using teledentistry by the PHVs Phase III: Oral screening and assessment of oral health needs was conducted by trained dentist . Analysis done for diagnostic accuracy testing for sensitivity, specificity, PPV, NPV	Strengths - Without a dentist’s direct examination, other qualified healthcare workers can conduct the dental disease screening. - As a result, less time, people resources, and dental visits are required. - Teledentistry can be effectively utilized for forming a sound diagnosis and treatment planning. Weakness - Only one PHV included - Only symptomatic areas were investigated - Presence of recall bias - Teledentistry imaging is insufficient to precisely detect all dental treatment needs required for each individual because pulp and periodontal status cannot be determined by imaging alone.	Santipipat et al. (26)
7	Survey	Malaysia	23 student participants for group discussions and 6 patients participated in in-depth interviews	Tele-education	The research included semi-structured group discussions with a qualitative approach and in-depth interviews with patients to phenomenologically explain the perspectives of the participants in the virtual community. With the consent of the participants, every session was videotaped. Using qualitative data analysis, the entire transcription of the recorded session was subjected to a thematic analysis using NVivoTM software.	Strengths - The results of this study will be helpful in creating and modifying clinical training programs in the future, giving instructors and students options in the event of a lockdown or other emergency. - The study’s qualitative methodology made it possible to comprehend the patients’ and students’ experiences adjusting to these new norms on a deeper level. The subtleties of human behavior were preserved while the subjects were questioned in their natural settings, in their own language, and according to their own terms. Weakness - Only patients and students who have participated in the virtual smoking sessions were included in the study. - The participants’ voluntary bias may have an impact on the generalizability of the results. Since those who were less devoted or who relapsed would not have accepted the invitation to participate, information bias may be present in the study.	Roslan et al. (27)
8	Survey	Indonesia	The study recruited 301 1st-, 2nd, & 3rd-year undergrad dental students from the Faculty of Dentistry at University Indonesia. The percentage of responses was 84.3% of which 85.1% of the participants were female.	Tele-education	The purpose of the questionnaire was to gauge how the students felt about the remote learning approach. With the exception of open-ended questions about the difficulties and rewarding experiences encountered while studying remotely and questions about the best strategies for remote learning, the response possibilities for the questionnaire items correspond to Likert-type scales. There were a total of 22 statements divided into four sections: (A) general details about the student’s gender, year of study, and GPA; (B) preference; (C) effectiveness; and (D) satisfaction with the learning process. Cronbach’s alpha was used to calculate the internal consistency reliability questionnaire score. Bivariate analysis and descriptive statistics computations were done. Logistic regression analysis was used to find the variables linked to the students’ choice for online learning. There was a 0.05 level of statistical significance.	Strength - Cronbach alpha of the questionnaire was 0.880, which is deemed acceptable. Weakness - The study was limited to a single university. - The intended 90% response rate was not met by the 84.3% response rate. - The study observed mainly on preclinical students as the main respondents, the study observed that clinical students in more advanced years of undergrad courses faced major challenges in learning during the pandemic years due to the complexity of subjects and the novelty of the online learning platforms. - This study’s questionnaire only assessed students’ perceptions. - The real amount of knowledge acquired by students and their perception of their learning are not strongly correlated.	Amir et al. (28)
9	Comparative Study	Thailand	37 pairs of participants participated in Study I and 34 pairs of participants participated in Study II.	Tele-education	In Study I which was conducted from October 2018 to February 2019, participants received in-person training and a 21-day chatbot course. In Study II from December 2021 to February 2022, participants received only daily chatbot programming for 30 days. The efficacy of the chatbots was evaluated in terms of knowledge gains, protection motivation theory-based attitudes of oral health care, and procedures for maintaining young children’s oral hygiene. A standardised questionnaire was utilised in both trials to collect data on socio demographic traits as well as baseline and follow-up oral health knowledge, attitudes, and practices. Questions on oral health knowledge included 11 items that addressed the following: when to start brushing, how often to brush, toothpaste with fluoride, brushing technique, and behaviour control for kids.	Strength - Cronbach alpha of the questionnaire was above 0.8 (has good internal consistency) Limitations - The data analysis has few limitations. - Both articles utilized a pre-post-methodology that might have a maturity bias. - The study might also have a vulnerability to examiner bias as the interview method and feedback period varied for both studies. - A self-administered online questionnaire was used and its validity could be prejudiced if subjects responded in a premediated manner, and a longer follow-up span could impact memory recall.	Pithpornchaiyakul et al. (29)
10	Cross-sectional study	Malaysia	631 adult patients from the Dental school of SEGI University	Teledentistry in general	The validated, self-administered online questionnaire had gathered information of patients’ background and dental-related history, accessibility to teledentistry services and their understanding of such services, as well as their willingness and obstacles in using them. The responses were collected from January 2020 to May 2021	Strength - Patient’s access to technology, acceptance and barriers in assimilating teledentistry in daily practice was explored. - The study had identified the necessity to train both patients and dentists in using teledentistry Weakness: - Results cannot be generalised as the sample size is not representative.	Zain et al. (30)

Data extraction, synthesis, study selection and quality assessment were done in a systematic manner and roles were divided among the research team. Two researchers from the study independently applied the metrics of Newcastle and Ottawa scale on the studies selected to be reviewed. Our analysis showed that studies authored by Maqsood et al. [25], Santipipat et al. [26], and Amir et al. [28] received the highest scores (9/10), indicating they are of superior quality according to the Newcastle-Ottawa Scale’s criteria for non-randomized studies. The articles by Rajendran et al. [21] and Soegyanto et al. [23] received a good score of 7/10. The remaining studies all scored 8/10, reflecting a high overall quality or selow risk of bias in their methodologies and reporting. (Table 3)

**Fig. 2:**
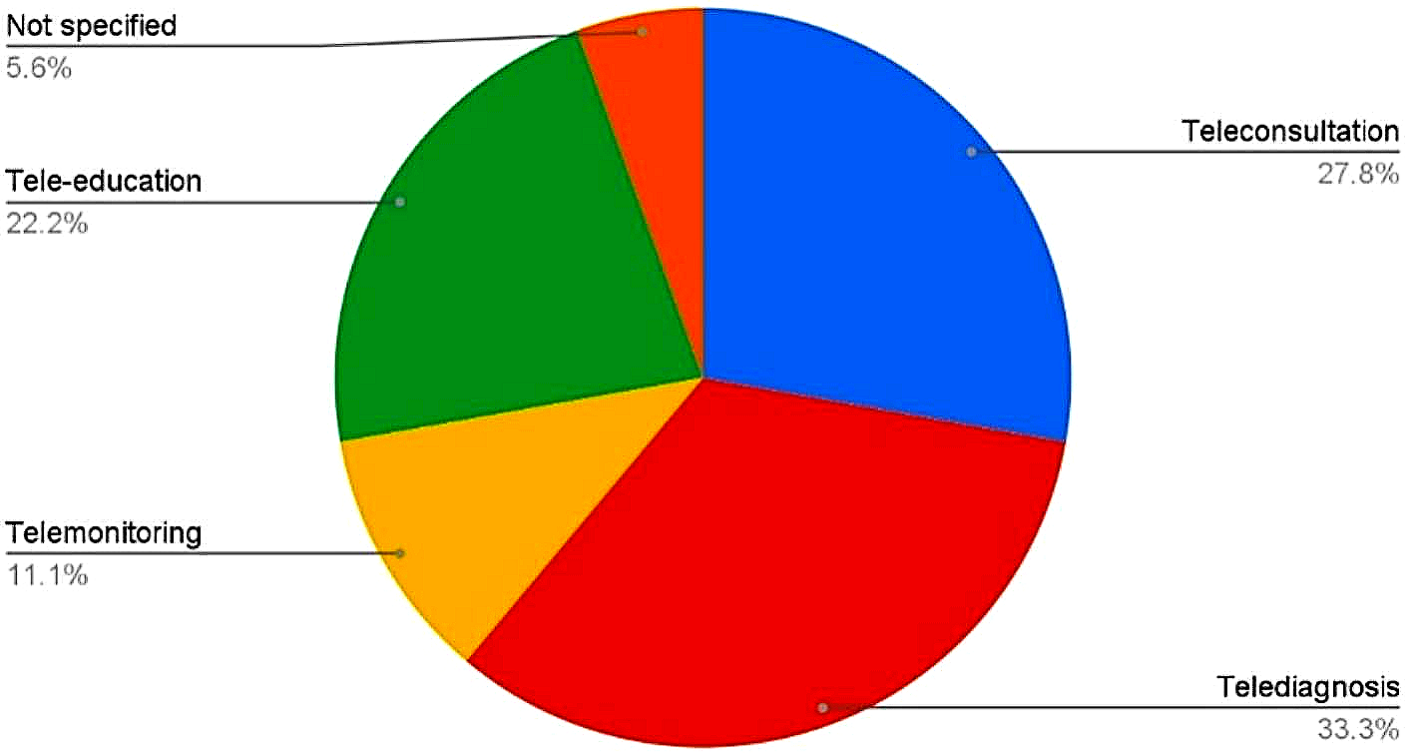
Sub domains of teledentistry

The current review has shown that teledentistry is being applied in various ways among ASEAN member states, ranging from teleconsultation [21–25], tele-diagnosis [21–26], telemonitoring [22, 23, 25] and tele-education [22, 25, 27–29], with tele-diagnosis being the most widely used service. (Fig. 2). Tele-diagnosis can be done in the synchronous or asynchronous form, otherwise known as “store-and-forward”. In the articles screened, the authors utilised the store-and-forward method, where the images were saved and sent to an oral pathologist for screening and diagnosing purposes [21, 26]. Tele-education is useful in public dental health where information can be disseminated to the public via various electronic platforms to educate them [27, 29]. The public benefited from teleconsultation services during the Covid-19 pandemic as they were able to access a dentist, albeit with limited services, due to the limitation of virtual consultations [24, 26, 27]. Telemonitoring was observed as the least used aspect of teledentistry in the ASEAN region. One of the articles [30] did not categorize the specific branch of teledentistry that it was trying to investigate and was too generic.

**Fig. 3:**
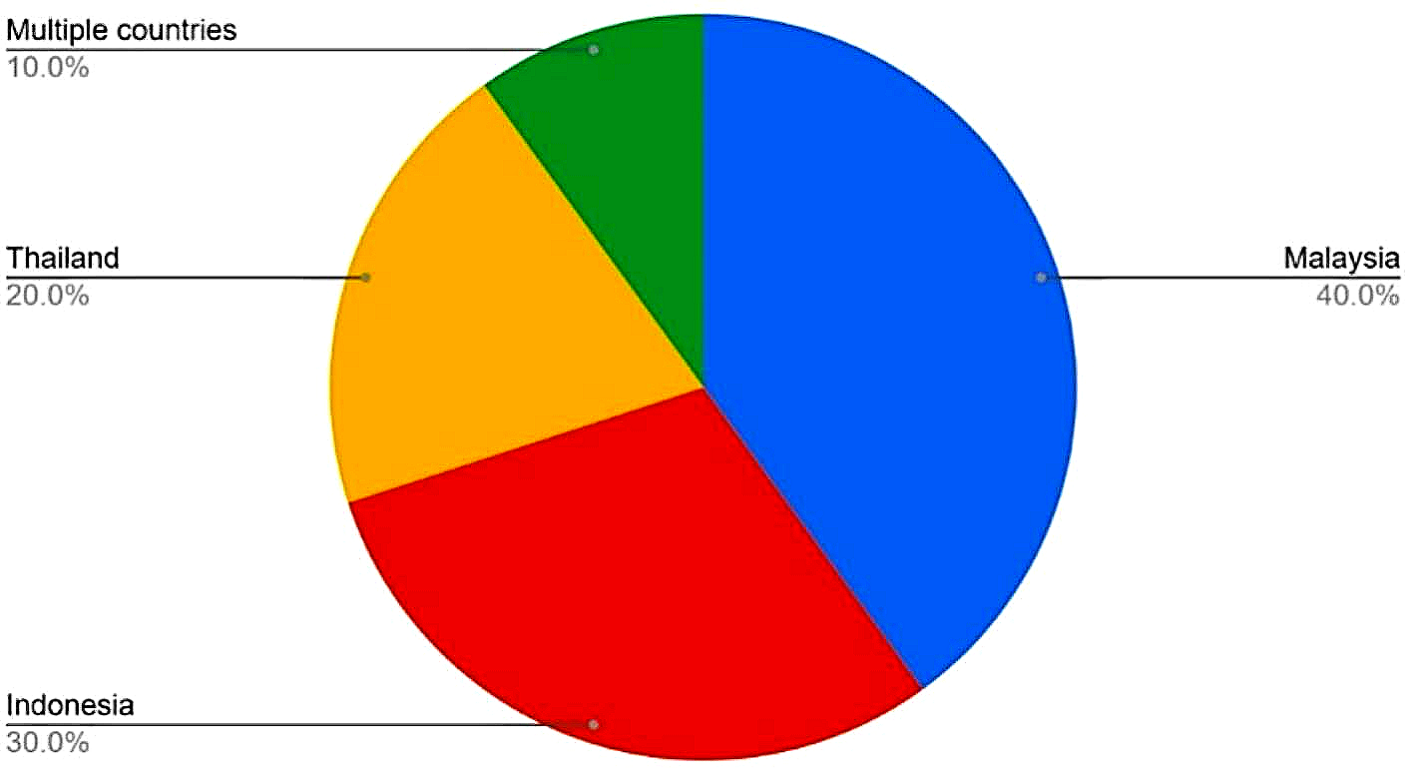
Teledentistry utilization in ASEAN countries

Among ASEAN member states, Malaysia [21, 22, 25, 27, 30] had been mentioned the most in peer-reviewed articles pertaining to teledentistry followed by Indonesia [23–25, 28], Thailand [26, 29] and Singapore [25], with Malaysia being mentioned five times, Indonesia four times, and twice for Thailand. Searches ran for Vietnam, Philippines, Myanmar, Cambodia, Laos, Brunei regarding teledentistry usage during the pandemic era revealed zero results. (Fig. 3)

**Fig. 4:**
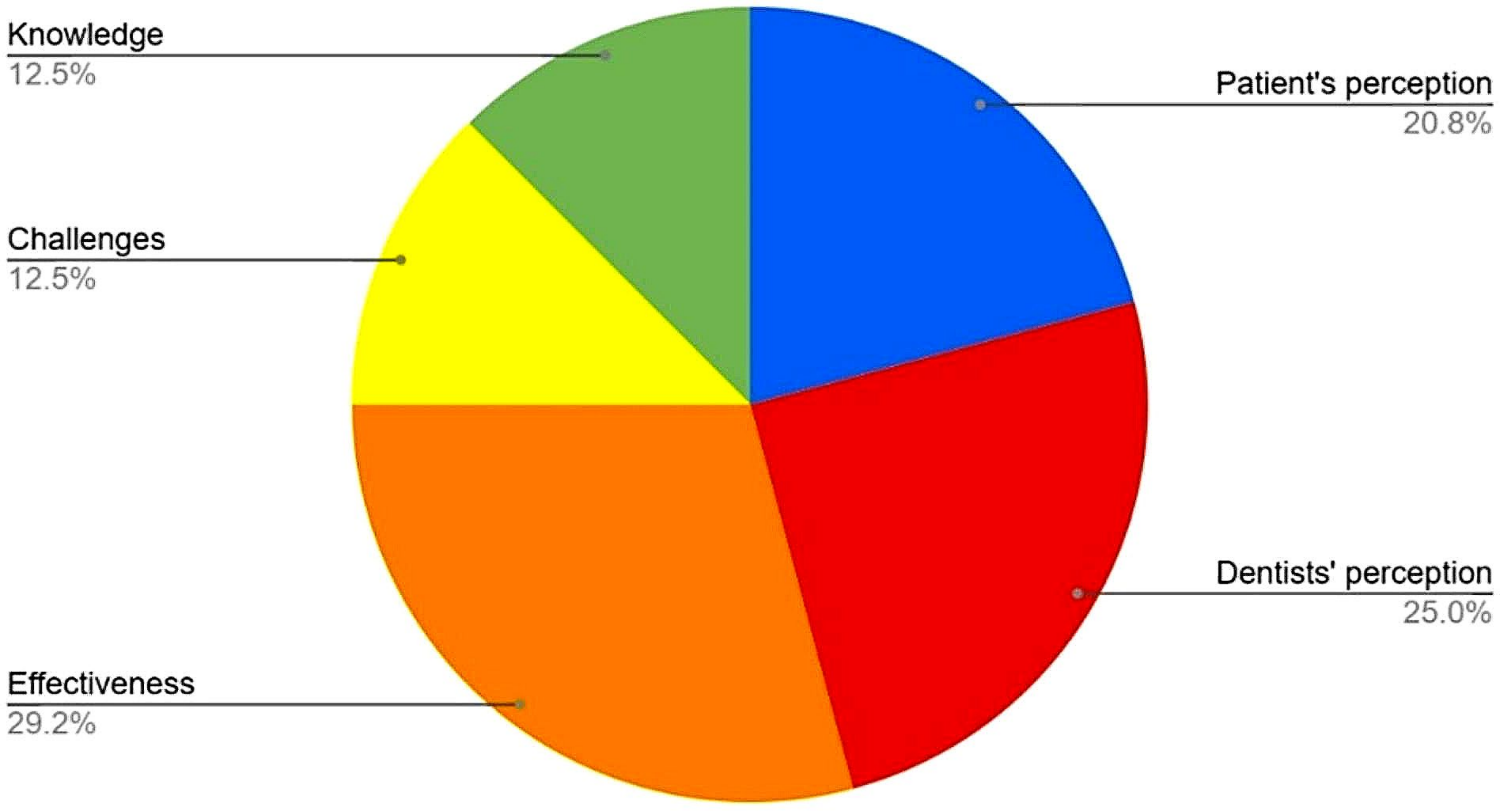
Teledentistry domains discussed in the included studies

Most of the included articles explored the effectiveness of teledentistry [22–26, 28, 29], as well as patients [23, 24, 27, 29], dentists’ and dental student’s [22, 23, 25, 27, 28] perceptions regarding e-dentistry services. Equal proportion of articles were related to knowledge [25, 28, 29] and challenges [22, 23] in applying teledentistry in daily practice. (Fig. 4). Most of the articles screened had a positive response and acceptability towards teledentistry. However, a number of survey participants from various studies were reluctant in adopting teledentistry due to various barriers identified, which will be discussed in the section below. The effectiveness of teledentistry was mainly gauged via surveys and a five-point Likert scale.

## Discussion

The ASEAN region is lagging behind western counterparts in embracing teledentistry in implementation and accessibility, and the trend remained consistent, during both pre & post pandemic era [12]. Our search entries for keywords on various databases uncovered very few articles, pertaining to teledentistry in ASEAN region before 2019. Notably, there exists a significant disparity between the acceptance of telehealth services, which are more readily adopted in the medical field compared to dentistry. During the pandemic, ASEAN, along with the rest of the world, witnessed a surge in virtual dentistry services [12]. While teledentistry is mainly used in oral medicine and dental public health context in the ASEAN region, its use is much broader in the Western world, where it is employed in not only in the aforementioned fields, but also in endodontics, prosthodontics, orthodontics, oral maxillofacial surgery, periodontology and paediatric dentistry [24, 25, 31–34].

Despite its increasing popularity, concerns about patient’s confidentiality, technical errors, poor image quality and accuracy of the diagnosis are quoted to be barriers to implementing teledentistry in daily practice [25–28]. In addition, the lack of training, appropriate infrastructure, poor digital literacy, poor awareness of teledentistry, the absence of a reimbursement system, and clear regulatory structures are among some of the common factors listed [23, 26–28, 30]. A scoping review on telemedicine guidelines and legislations revealed that Singapore, Malaysia, Indonesia, Thailand and Vietnam have laws to regulate telemedicine and telepharmacy services, but none that are specific to teledentistry [35]. In addition, according to Marya et al. [12], there are currently no legislations, concerning teledentistry in the ASEAN region. Due to these factors, teledentistry is mainly used as an adjunctive tool and cannot substitute conventional in-person dental visits.

It cannot be denied that teledentistry comes with many benefits, chief amongst them is convenience to patients, who often have limited accessibility to healthcare services, particularly in remote and low resource areas [17, 36]. As ASEAN countries are all still developing, there are many regions that have poor access and lack of resources. Accessibility was critical, particularly when movement restrictions were in place during the Covid-19 pandemic. Teledentistry, can be very helpful in breaking the barriers of accessibility by providing remote dental screening to minimise unnecessary dental visits, prior to scheduling an appointment with a dental practitioner or proceeding with the referral process to a specialist. Furthermore, during COVID-19 period, teledentistry played a vital role in reducing the risk of COVID-19 transmission by minimising in-person visits, which reduces exposure of patients’ and dental staff’ to the virus [12].

Advances in information technology have increased the effectiveness of teledentistry manifolds and teledentistry based solutions have shown effectiveness in minimising direct contact between patient and dentist. A randomised controlled crossover study conducted in Thailand, on using intraoral cameras to facilitate a dental screening programme [26] showed that intraoral cameras used by trained health personnels can be helpful in diagnosing oral diseases and dental caries without requiring a direct examination by a dental professional. This technology proved to be a highly a valuable asset for dental screening and diagnostic purposes, enabling preliminary investigations and treatment planning before patients are referred to a dental specialist. Moreover, it minimised working time, number of dental visits, human resources and cost of the patient and the dentist. However, the screening should be carried out by trained health personnels, and proper guidelines should be provided to them for accurate image recording. Another crossover study involving the development of the web interface MEMOSA in Malaysia [21] underscored the potential of a web interface as a supplementary tool for accurately diagnosing and categorising patients in need of referrals and emergency care, particularly for cancer surveillance.

A comparative research study conducted in Thailand during the COVID-19 pandemic reported a significant enhancement in patient education [29]. In this study, parents and caregivers were directed to utilise their mobile devices to access a chatbot via Facebook Messenger, which subsequently disseminated dental knowledge to them over the course of 21–30 daily sessions. The educational content encompassed crucial aspects, including the importance of supervised brushing, correct tooth brushing techniques, the recommended frequency of daily brushing, and the application of fluoridated toothpaste, all delivered exclusively through the chatbot interface. To minimize face-to-face interactions, instructional video clips were employed to demonstrate toothbrushing techniques, and a follow-up questionnaire was administered for assessment purposes. Additionally, children’s oral hygiene was evaluated through direct examination by a dentist, primarily to assess visible plaque. The outcomes of this study underscored a notable improvement in parent and caregiver education and showed efficacy in significant plaque reduction. More importantly, positive feedback from parents and caregivers also strongly suggests that chatbots have the potential to effectively substitute for in-person training in the realm of oral hygiene education.

Furthermore, a qualitative study on the experiences and perceptions of Malaysian dental undergraduates and their patients has proven that virtual counselling is beneficial in achieving smoking cessation [27]. In this article, the authors explored on many themes which included, (1) General opinions and experiences, (2) Content of virtual consultations (VCs), (3) Remote access to counselling, (4) Patient-clinician relationships, (5) Technical issues, (6) Changes after VCs, and (7) Future application. Most of the dental students and patients found virtual counselling to be convenient and acceptable as a treatment option for this matter. It is particularly beneficial for this case of counselling, where some patients find it unnecessary to travel all the way to the clinic just for a short counselling session. Moreover, it is beneficial for patients who are busy with their work and find it hard to attend a counselling session during working hours. One of the major setbacks of this virtual counselling is that the patients may not be truthful in answering the questions. Also, since a Smokerlyzer test was not conducted, and the periodontal status cannot be determined virtually, the authors were unable to determine if there were any objective improvements after tobacco cessation. The same scenario is also reflected in oral medicine where in-office visit is needed to assess healing in postoperative oral potentially malignant diseases cases [24].

The COVID-19 pandemic has not only impacted dental healthcare services for patients and practitioners but has also had significant repercussions for undergraduate dental students and practicing dentists who traditionally acquire new dental knowledge and skills through in-person lectures, workshops, and seminars [28, 37]. Tele-education has emerged as an innovative solution, offering an alternative learning experience to remotely enhance dental knowledge through technology, all while reducing the need for face-to-face interactions during the pandemic. A survey was conducted in Indonesia to assess the perspective of undergraduate dental students from Faculty of Dentistry Universitas Indonesia on classroom and distance learning methods [28]. The questionnaire consisted of four parts: (A) General information on the student’s gender, year of study and GPA, (B) Preference, (C) Effectiveness, and (D) Learning satisfaction. Additionally, open-ended questions were included to solicit insights into challenges encountered and positive experiences gained during online learning. The results indicated that first-year undergraduate students exhibited a greater inclination toward distance learning compared to their senior counterparts. They expressed a preference for remote discussions and clarification sessions. Students believed that online classes afforded them more self-directed learning opportunities compared to traditional in-person classes. Nevertheless, students acknowledged facing challenges with distance learning, including the need to adapt to a new learning method, difficulties in maintaining focus when using digital devices, and a reduction in social interaction and personal contact with peers and lecturers. These challenges had an impact on the quality of their learning experiences and created additional pressure during their educational journey.

### Strengths and limitations

The authors recognize the strengths and limitations of this systematic review. We have identified several limitations. Due to the scarcity of research articles in the ASEAN region, generating a sustainable conclusion becomes challenging. Furthermore, only a fraction of ASEAN member states had published articles on teledentistry during the Covid-19 pandemic.

Additionally, volunteer bias is present in two of the retrieved papers [23, 27] as the survey participants were not randomly chosen, but consisted of people who joined willingly. Self-reported bias [25, 26, 29] and non-response bias [22] were also prevalent among the articles screened.

Most of the research articles conducted in ASEAN explored the perceptions of patients. As valuable as these studies are, they might not be as adequate to be used as a marker to assess the effectiveness of the actual clinical efficacy of teledentistry in treating patients. The review clearly underscores the need for more research as well as application of teledentistry based solution in the ASEAN region. We have observed a dearth of research, application but also a massive opportunity for the region to invest more on teledentistry research and application. We have also identified a lack of legislation and guidelines in several Asean countries which also needs addressed.

## Conclusion

Teledentistry is a practical way to screen and educate the public especially those with poor access such as in remote or unprivileged areas as well during times of crisis, where face-to-face contact is limited. While it may be used to screen patients, it cannot replace in-office visits to reach a diagnosis. Since most ASEAN states do not have a legislative document to regulate the use of teledentistry, there is a need for a standardised legislation which states the requirements for teledentistry, practices that are allowed to utilise teledentistry, as well as the reimbursement system. It is hoped that with such laws, it may encourage the widespread use of teledentistry among ASEAN dentists.

## Data Availability

The datasets used and/or analysed during the current study available from the corresponding author on reasonable request.

## Declarations

The study was approved by International Medical Universities Ethical committee with the project number of BDS I-01-2023(01).
